# Quantification of the Arctic Sea Ice‐Driven Atmospheric Circulation Variability in Coordinated Large Ensemble Simulations

**DOI:** 10.1029/2019GL085397

**Published:** 2020-01-17

**Authors:** Yu‐Chiao Liang, Young‐Oh Kwon, Claude Frankignoul, Gokhan Danabasoglu, Stephen Yeager, Annalisa Cherchi, Yongqi Gao, Guillaume Gastineau, Rohit Ghosh, Daniela Matei, Jennifer V. Mecking, Daniele Peano, Lingling Suo, Tian Tian

**Affiliations:** ^1^ Woods Hole Oceanographic Institution Woods Hole MA USA; ^2^ Sorbonne Université, CNRS/IRD/MNHN, UMR LOCEAN Paris France; ^3^ National Center for Atmospheric Research Boulder CO USA; ^4^ Fondazione Centro Euro‐Mediterraneo sui Cambiamenti Climatici Bologna Italy; ^5^ Istituto Nazionale di Geofisica e Vulcanologia Bologna Italy; ^6^ Nansen Environmental and Remote Sensing Center and Bjerknes Center for Climate Research Bergen Norway; ^7^ Nansen‐Zhu International Research Center, Institute of Atmospheric Physics Chinese Academy of Sciences Beijing China; ^8^ Max Planck Institute for Meteorology Hamburg Germany; ^9^ Ocean and Earth Science, National Oceanography Centre Southampton University of Southampton Southampton UK; ^10^ National Oceanography Centre Southampton Southampton UK; ^11^ Danish Meteorological Institute Copenhagen Denmark

## Abstract

A coordinated set of large ensemble atmosphere‐only simulations is used to investigate the impacts of observed Arctic sea ice‐driven variability (SIDV) on the atmospheric circulation during 1979–2014. The experimental protocol permits separating Arctic SIDV from internal variability and variability driven by other forcings including sea surface temperature and greenhouse gases. The geographic pattern of SIDV is consistent across seven participating models, but its magnitude strongly depends on ensemble size. Based on 130 members, winter SIDV is ~0.18 hPa^2^ for Arctic‐averaged sea level pressure (~1.5% of the total variance), and ~0.35 K^2^ for surface air temperature (~21%) at interannual and longer timescales. The results suggest that more than 100 (40) members are needed to separate Arctic SIDV from other components for dynamical (thermodynamical) variables, and insufficient ensemble size always leads to overestimation of SIDV. Nevertheless, SIDV is 0.75–1.5 times as large as the variability driven by other forcings over northern Eurasia and Arctic.

## Key Points

- Arctic sea ice variations drive ~1.5% of boreal winter Arctic SLP variance during 1979–2014 in coordinated 130‐member AGCM experiments
- An accurate quantification of the impacts of Arctic sea ice‐driven SLP variability requires an ensemble size greater than 100 members
- Insufficient ensemble size always leads to an overestimation of the Arctic sea ice‐driven variance within and outside Arctic

## 1 Introduction

The rapid loss of Arctic sea ice since the late 1970s has been observed by routine satellite missions (Stroeve & Notz, 2018) and shown to profoundly affect weather, environment, and ecosystem in the Arctic (Gerland et al., 2019; Meier et al., 2014). Many studies have argued that the Arctic sea ice melting can also exert far‐reaching effects on midlatitude weather extremes and climate variability (Honda et al., 2009; Petoukhov & Semenov, 2010; Francis & Vavrus, 2012; Cohen et al., 2014; Kim et al., 2014; Mori et al., 2014; Overland et al., 2015; Kretschmer et al., 2016; Overland et al., 2016; and many others), although this topic has been controversial (e.g., Blackport et al., 2019; Overland et al., 2016; Screen & Blackport, 2019). Global climate model simulations show a full spectrum of atmospheric circulation responses to Arctic sea ice loss. For example, some atmospheric general circulation model (AGCM) experiments that prescribe reduced Arctic sea ice conditions show a negative Northern Annular Mode (or Arctic Oscillation; Thompson & Wallace, 1998) response in boreal winter (Seierstad & Bader, 2009; Peings & Magnusdottir, 2014), while others show a weak or opposite response (Cassano et al., 2014; Screen et al., 2014; Singarayer et al., 2006; Strey et al., 2010). Such inconsistencies call for improved understanding of the causality of the Arctic‐midlatitude linkages and underlying mechanisms.

The reasons for these inconsistencies among the modeling studies likely arise from different model configurations, model dependency, varying strengths, or geographic patterns of the Arctic sea ice forcing used to drive AGCMs, different model sensitivities to Arctic sea ice changes, and/or dependencies on the different background climate states (Cohen et al., 2018; Li et al., 2018; Ogawa et al., 2018; Screen et al., 2018). Screen et al. (2014) highlighted the need for large ensemble simulations to robustly separate the atmospheric response to Arctic sea ice changes from natural atmospheric internal variability (or noise). Such intrinsic variability is certainly capable of masking out any local and remote impacts of Arctic sea ice‐forced circulation changes by lowering the signal‐to‐noise ratio in climate model simulations. In response, the scientific community has recently proposed to conduct coordinated large ensemble AGCM experiments, in which experimental design typically requires use of a common Arctic sea ice distribution and concentration imposed over a specified period of time (Screen et al., 2018; Smith et al., 2019). One such large ensemble set of experiments using state‐of‐the‐art AGCMs has been recently coordinated under the umbrella of the Blue‐Action Project, an international collaboration primarily supported by the European Union (blueaction.eu), to better understand the atmospheric circulation response to the observed Arctic sea ice variability. This study uses simulations from seven modeling groups participating in this project, with a total of 130 ensemble members, to quantify the atmospheric circulation response to Arctic sea ice variability during the 1979–2014 period.

## 2 Data Sets and Methods

### 2.1 Coordinated Multimodel AGCM Experiments

Seven AGCMs used in this study are listed in Table S1. Following the protocol developed by the Blue‐Action Project, global daily ¼ degree sea surface temperatures (SSTs) and sea ice concentrations (SICs) during the 1979–2014 period from U.K. Met Office Hadley Centre Sea Ice and SST Version 2.2.0.0 (Kennedy et al., 2017; Rayner et al., 2003; Titchner & Rayner, 2014) are used to force the AGCMs. This data set was developed in the framework of the HighResMIP panel of Coupled Model Intercomparison Project Phase 6 (CMIP6) protocol (Haarsma et al., 2016). The first set of experiments uses time‐varying global SST and SIC fields, which is called “ALL” to denote its inclusion of all forcings hereafter, whereas the second set replaces the SIC field in the Northern Hemisphere by its climatological (1979–2014 average) daily values while keeping the time‐varying SIC in the Southern Hemisphere and time‐varying SST globally, which is called “SIC_clim_” to denote the exclusion of Arctic SIC forcing from others.

A series of SST and SIC adjustments following Hurrell et al. (2008) are performed to have consistent SST and SIC values. For ALL, a four‐step adjustment method is applied as follows:

1. Set minimum SST to −1.8 °C.
2. Set SST to −1.8 °C, if SIC ≥90%.
3. Set SIC to 0, if SST > 5 °C.
4. If SIC <90%, we calculate SST_max_ = 9.328 * (0.729‐SIC^3^) − 1.8), an empirical distribution function derived based on Figure 4 of Hurrell et al. (2008). If SST > SST_max_, we reduce the SIC by solving above equation assuming SST_max_ = SST.

For SIC_clim_, we use daily SST climatology (1979–2014 average) wherever climatological SIC is greater than 0, then repeat the above Steps 2 to 4 to modify as needed.

The greenhouse gas (GHG) emissions and aerosol forcings during the 1979–2014 period are specified in both ALL and SIC_clim_ following CMIP6 (Eyring et al., 2016; Haarsma et al., 2016). Each participating group has conducted 10 to 30 ensemble members for each experiment, resulting in a total of 130 members (Table S1). It is noted that not every group followed all the steps of the protocol (Table S1), but the results using the subset of AGCM simulations that followed the protocol exactly are very similar and do not alter the conclusion in any significant way. This study uses monthly data for the 1979–2014 period in the boreal winter months, when the atmospheric response to Arctic sea ice change was examined in previous studies (e.g., Deser et al., 2010; Porter et al., 2012; Screen et al., 2013). Before analysis, we take December‐January‐February average (DJF hereafter) for each variable.

### 2.2 Variance Decomposition

Using the multimodel large ensemble of ALL and SIC_clim_, we decompose the DJF total variability of each variable into its components: the internal atmospheric noise, the Arctic sea ice‐driven variability, and the variability forced by all the other boundary and radiative forcings, including SST, external forcings such as GHG, and sea ice in the Southern Hemisphere (the last component will be simply called SST/GHG‐driven variability, hereafter). In the following analyses, for a given variable *X* at a specific longitude‐latitude grid point (i.e., *X* has two dimensions: ensemble dimension and time dimension), $\bar{X}$ denotes the average of *X* over the ensemble dimension (i.e., $\bar{X}$ has time dimension left only), while *VAR(X)* denotes the variance of *X* over the time dimension (i.e., *VAR(X)* has ensemble dimension left only). The total variability is simply calculated from the ALL ensemble, *X*_*ALL*_. The internal atmospheric variability (or noise) is estimated by subtracting the ensemble mean of ALL at each time step from the total variability, $X_{\mathrm{ALL}}-\bar{X_{\mathrm{ALL}}}$. Next, the SST/GHG‐driven variability is estimated from the ensemble average of SIC_clim_, $\bar{X_{{\mathrm{SIC}}_{\text{clim}}}}$. Finally, the Arctic SIC‐driven (called SIC‐driven hereafter) variability is estimated from the difference between the ensemble averages of ALL and SIC_clim_, $\bar{X_{\mathrm{ALL}}}-\bar{X_{{\mathrm{SIC}}_{\text{clim}}}}$.

This decomposition is validated using the variance formula, that is, the variance of the total variability is equal to the sum of the variances and two times the covariances of the subcomponents. Specifically, the variance, *VAR*, and the covariance, *COVAR*, terms are defined as follows:

(1) $$\text{Total variability}:\;\bar{\mathrm{VAR}\left(X_{\mathrm{ALL}}\right)},$$

(2) $$\text{Internal atmospheric variability}:\;\bar{\mathrm{VAR}\left(X_{\mathrm{ALL}}-\bar{X_{\mathrm{ALL}}}\right),}$$

(3) $$\frac{\mathrm{SST}}{\mathrm{GHG}}-\text{driven variability}:\;\mathrm{VAR}\left(\bar{X_{{\mathrm{SIC}}_{\text{clim}}}}\right),$$

(4) $$\mathrm{SIC}-\text{driven variability}:\;\mathrm{VAR}\left(\bar{X_{\mathrm{ALL}}}-\bar{X_{{\mathrm{SIC}}_{\text{clim}}}}\right),$$

(5) $$\mathrm{SIC}-\frac{\mathrm{SST}}{\mathrm{GHG}}-\text{driven covariability}:2\text{COVAR}\left(\bar{X_{\mathrm{ALL}}}-\bar{X_{{\mathrm{SIC}}_{\text{clim}}}},\bar{\;X_{{\mathrm{SIC}}_{\text{clim}}}}\right).$$

**Figure 1:**
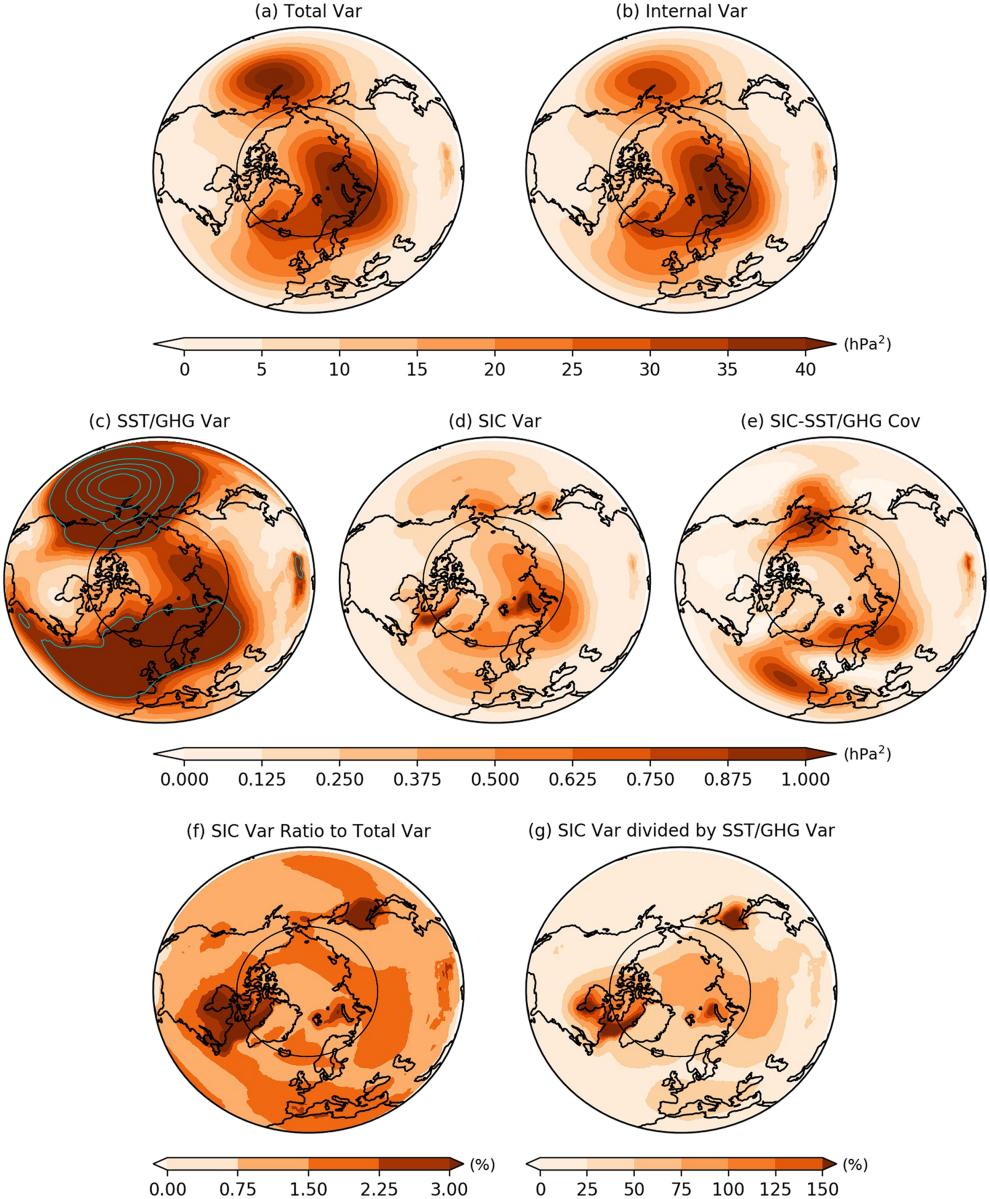
Variance decomposition for the Northern Hemisphere (20–90°N) DJF‐mean SLP from the 130‐member multi‐model ensemble. (a) Total variance decomposed into the variances of (b) internal atmospheric variability, (c) SST/GHG‐driven variability, (d) SIC‐driven variability, and (e) covariance between SIC‐driven and SST/GHG‐driven components. Note that the values shown in panel (e) are multiplied by −1 to aid comparison with other panels. The cyan contour lines in (c) denote values larger than 1 hPa^2^ with interval 2 hPa^2^. (f) The ratios of the SIC‐driven variance (d) to the total variance (a) in percentage. Panel (g) shows the ratio between the SIC‐driven and SST/GHG‐driven components, that is, (d) divided by (c) in percentage. The black circle corresponds to 65°N to denote the Arctic Circle.

**Figure 2:**
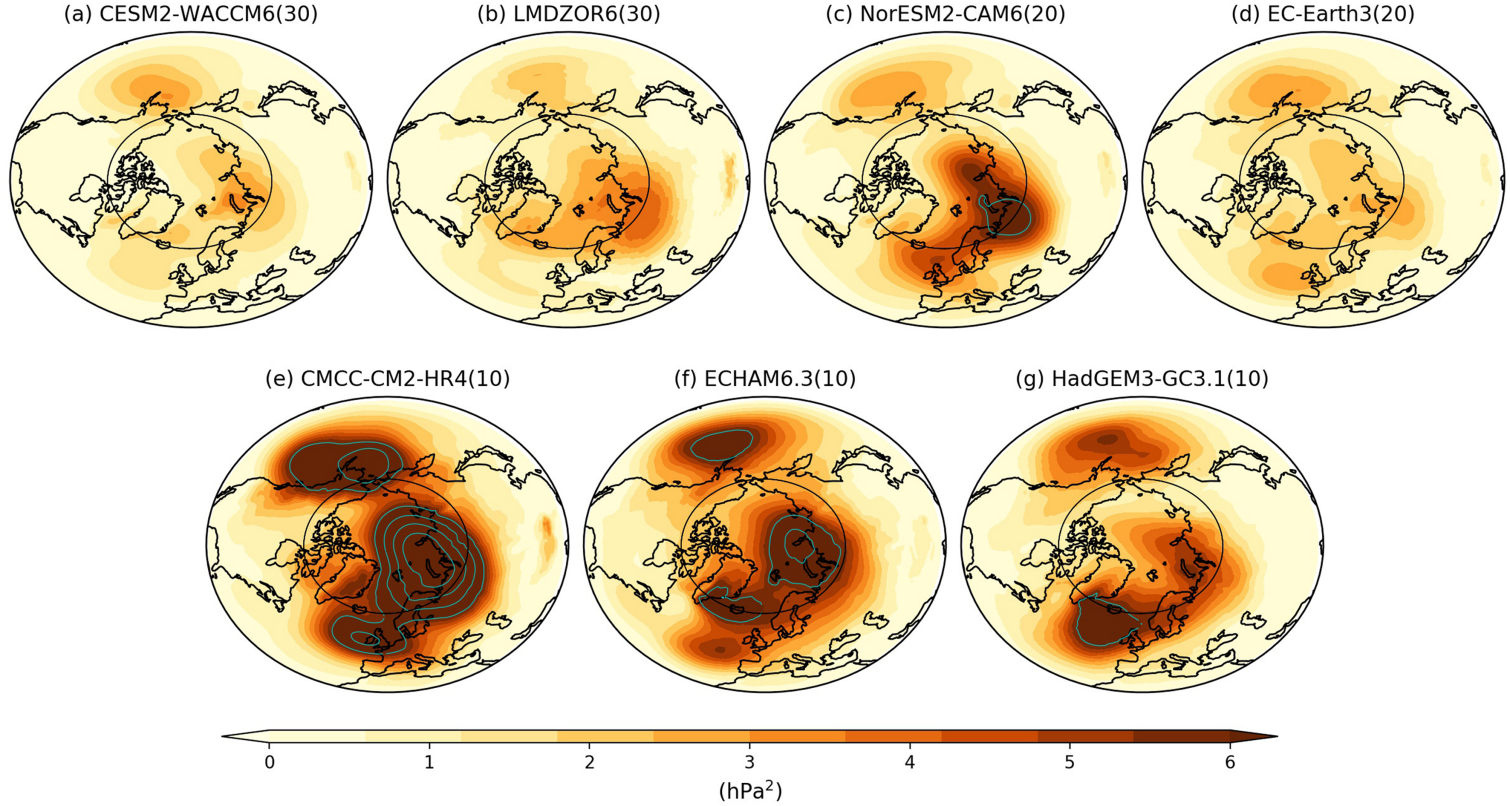
Arctic SIC‐driven variances of DJF SLP for (a) CESM2‐WACCM6, (b) LMDZOR6, (c) NorESM2‐CAM6, (d) EC‐Earth3, (e) CMCC‐CM2‐HR4, (f) ECHAM6.3, and (g) HadGEM3‐GC3.1. The number in the parenthesis denotes ensemble size in each AGCM. The cyan contour lines denote values larger than 6 hPa^2^ with interval 2 hPa^2^. The black circle corresponds to 65°N to denote the Arctic Circle.

**Figure 3:**
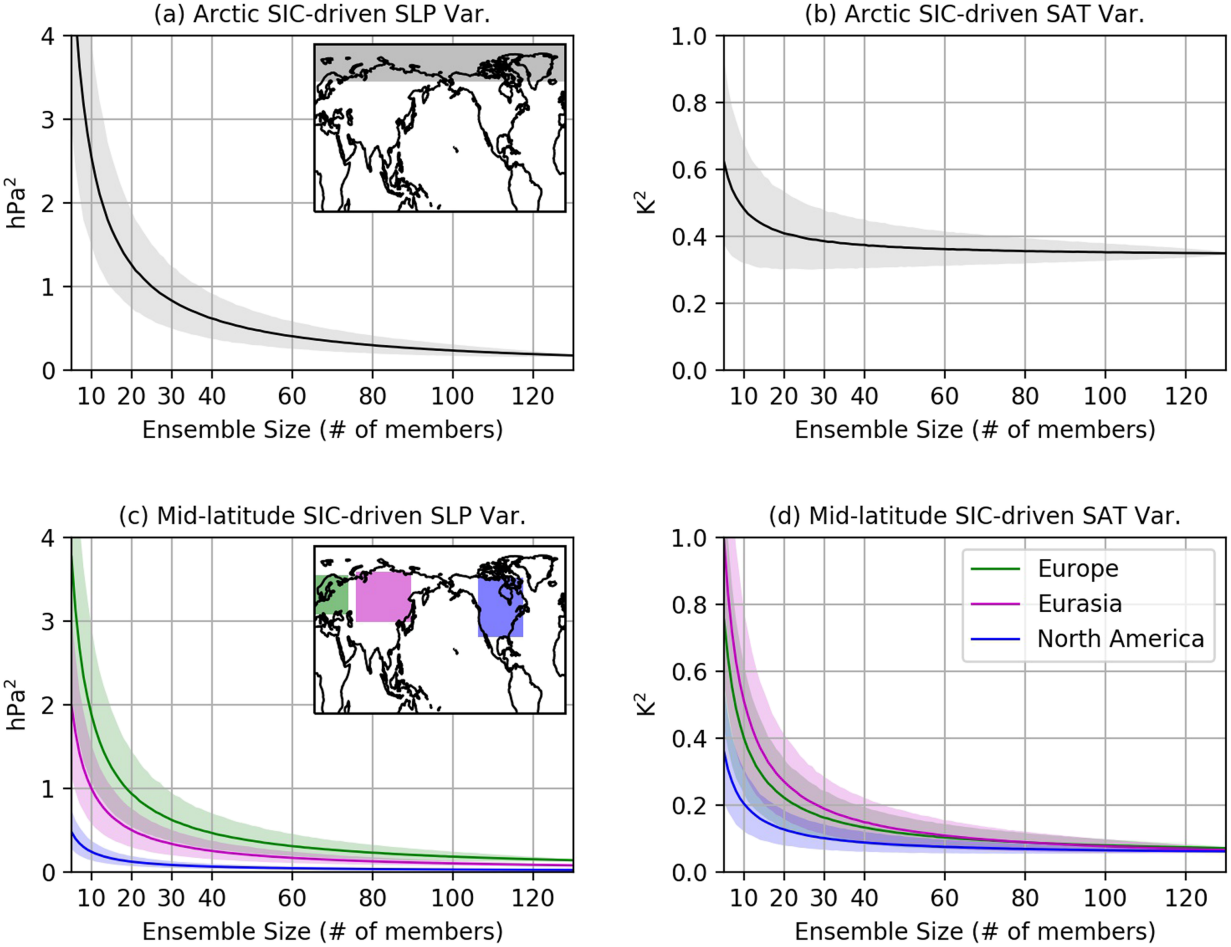
Ensemble size dependency for the SIC‐driven variance estimates. The top row is for the Arctic‐averaged (65–90°N) DJF SLP (a) and SAT (b). For each given ensemble size, the ensemble members are randomly sampled without replacement 10,000 times. The shadings indicate the 95th percentile range from 10,000 random selection and the average is plotted with the solid curves. (c, d) Same as (a) and (b) but for three selected midlatitude domains. The regions for calculating area average values are shaded with corresponding colors in the subpanels of (a) and (c). The midlatitude European domain is 45–71°N and 0^–^50°E (green patch in c), Eurasian domain 40–73°N and 60–140°E (magenta patch in c), and North American domain 30–69°N and 130–60°W (purple patch in c).

**Figure 4:**
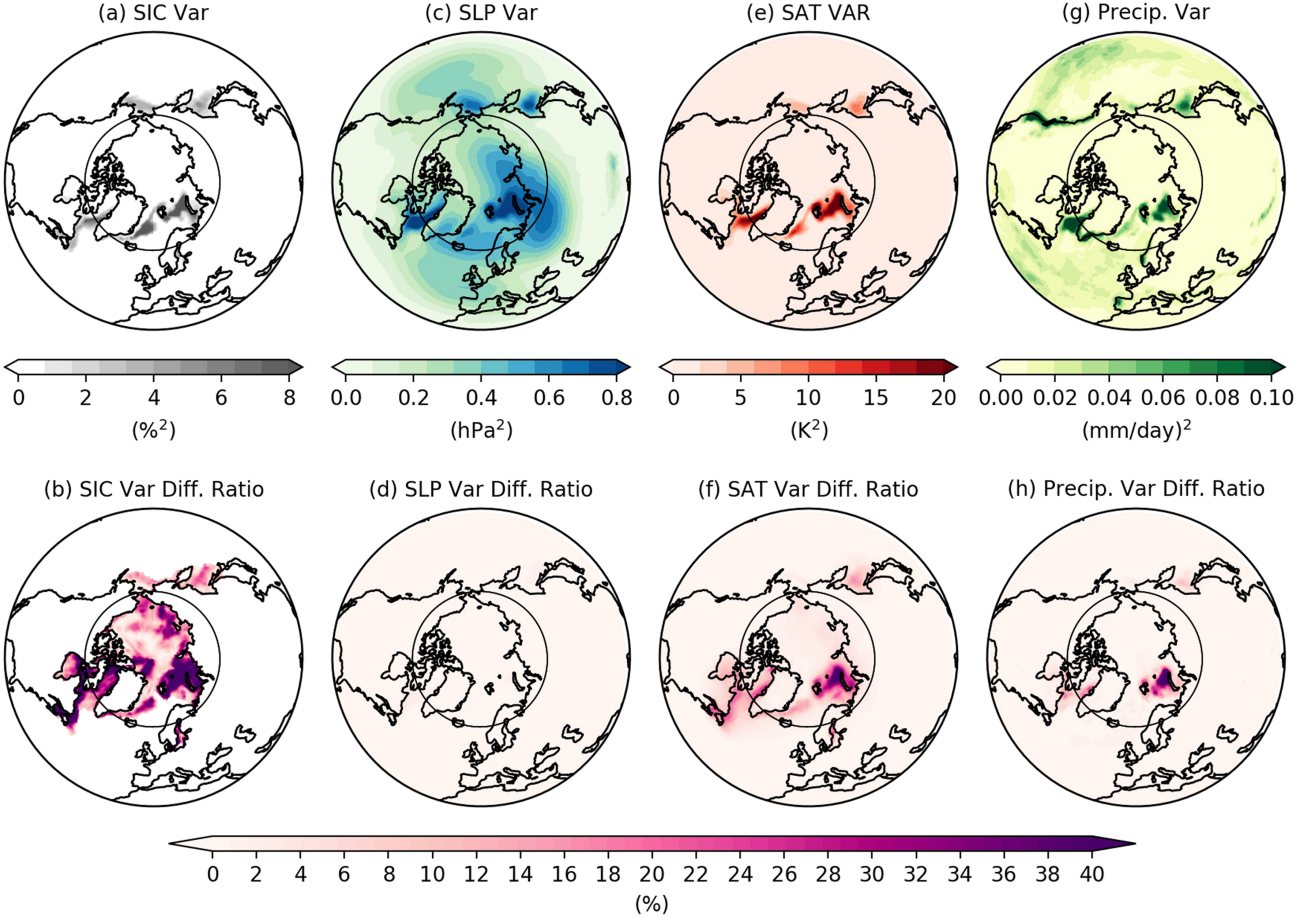
(a) Variance of the observed SIC, which is prescribed in the ALL. (b) Ratio of (a) minus its counterpart without quadratic trend to (a), that is, the percentage of the total SIC variance due to quadratic trend. (c, e, and g) Same as (a) but for the SIC‐driven variability of SLP, SAT, and total precipitation (large‐scale plus convective precipitation) with 130 members, respectively. (d) The percentage of the total variance due to the quadratic trend in SIC‐driven SLP variability, calculated as (c) minus its counterpart without quadratic trend divided by the total variance of SLP. (f and h) Same as (d) but for the SAT and total precipitation. The black circle corresponds to 65°N to denote the Arctic Circle.

The residual term, given by

$$\text{Residual}=\left(1\right)-\left(2\right)-\left(3\right)-\left(4\right)-\left(5\right)=\bar{2\text{COVAR}\left(X_{\mathrm{ALL}}-\bar{X_{\mathrm{ALL}}},\bar{X_{{\mathrm{SIC}}_{\text{clim}}}}\right)}+\bar{2\text{COVAR}\left(X_{\mathrm{ALL}}-\bar{X_{\mathrm{ALL}}},\bar{X_{\mathrm{ALL}}}-\bar{X_{{\mathrm{SIC}}_{\text{clim}}}}\right)},$$

is very small and can be neglected. For sea level pressure (SLP), its maximum residual variance (the ratio to total variance) over the Northern Hemisphere is about 10^−4^ hPa^2^ (10^−2^%; not shown), much smaller than that of the SIC‐driven variability. For the surface air temperature, the maximum residual variance (the ratio) is also about 10^−4^ K^2^ (10^−2^%; not shown). Such small residuals indicate that the variance decomposition works well. In Figures 1, 2, 4, and S1 in the supporting information, we perform the variance decomposition at each Northern Hemisphere grid point to obtain spatial maps of SIC‐driven variability (and other variance components). In Figures 3 and S2, we first take area‐averaged values over the domain of interest and then perform the variance decomposition.

## 3 Results

We first examine the DJF SLP variability in the AGCM experiments, as the DJF SLP is a manifestation of weather and large‐scale atmospheric circulation variability in boreal winter. Fig. 1 illustrates the variance decomposition of the DJF SLP variability in the Northern Hemisphere (20–90°N) based on the ALL and SIC_clim_ 130‐member ensembles using the seven participating models.

The total variability exhibits two major centers of action with high variance reaching 40 hPa^2^ (Figure 1a). The first center is in the North Pacific, where Aleutian Low system dominates; the second in the Barents‐Kara Seas and northern Eurasia, reflecting in part the Ural blocking system. Slightly weaker local maximum variances (~30 hPa^2^) also appear over Iceland, reflecting the Icelandic Low system. The variability of internal atmospheric noise shows a similar spatial structure with slightly weaker amplitudes (Figure 1b) and explains overall more than 70% of the total variability.

The SST/GHG‐driven variability has the strongest variance with ~10 hPa^2^ in the Aleutian Low region (cyan contour lines in Figure 1c). This likely reflects the teleconnections associated with El Niño–Southern Oscillation in the tropical Pacific at interannual timescale (Alexander et al., 2002; Wallace & Gutzler, 1981), and Pacific decadal variability at longer timescale (Mantua et al., 1997). The SST/GHG forcing also affects the SLP variability in the Icelandic Low region and over Eurasia, which may be related to the North Atlantic SST variability and its downstream effects (Gastineau & Frankignoul, 2015; Luo et al., 2016; Sato et al., 2014).

The Arctic SIC‐driven SLP variability has overall smaller variances peaking over the Barents‐Kara Seas (~1 hPa^2^) and part of northern Eurasia (~0.75 hPa^2^) (Figure 1d), which explain ~3% and ~2% of the total variance, respectively (Figure 1f). Local maxima can be found over the Labrador Sea and the Sea of Okhotsk (~3%, Figure 1f), possibly caused by large sea ice variations in these regions. We further compare the sea ice‐driven and the SST/GHG‐driven components by examining the ratio between the two, that is, SIC‐driven SLP variance divided by the SST/GHG‐driven variance (Figure 1g), which shows that the former explains nearly 150% of the variance explained by the latter over the Labrador Sea, Hudson Bay, Barents Seas, and Sea of Okhotsk, nearly the same amount of total variance over Kara Sea, and ~75% over parts of northern Eurasia. Over the North Atlantic, Europe, and East Asia, the SIC‐driven variability is about 25–50% of that of the SST/GHG‐driven variability. Although the winter SIC‐driven SLP variability is much smaller than the SLP internal variability, it is larger or comparable to that driven by other forcings within the regions where sea ice variation is large and over parts of northern Eurasia and North America.

The covariance due to SIC‐driven and SST/GHG‐driven components exhibits maximum values over the Bering Strait and Alaska, near the midlatitude jet streams in the North Pacific and North Atlantic, and northeastern Europe (Figure 1e; note that the values are multiplied by −1 for better comparison to other components). The covariance term reflects the covariability between the Arctic SIC and SST/GHG forcings, possibly due to their nonlinear interactions (e.g., Han et al., 2016).

We next compare the spatial patterns of Arctic SIC‐driven SLP variability in the seven AGCMs in Figure 2. The overall patterns are broadly similar across the models with centers of action over the Northeast Pacific, Barents‐Kara Seas, and Northeast Atlantic. The patterns are also similar to 130‐member ensemble average (Figure 1d). This suggests that the SIC forcing does not create new large‐scale circulation pattern but projects onto the leading mode of internal variability, as discussed in previous studies (Deser et al., 2004; Peng & Robinson, 2001). However, the magnitudes among AGCMs differ substantially, with a general tendency of larger variance for the models with a smaller ensemble size: CESM2‐WACCM6 and LMDZOR6 with 30 members have similar variances over centers of action, which are ~50% of those in CMCC‐CM2‐HR4 and ECHAM6.3 with 10 members each. Furthermore, any single model ensemble exhibits larger amplitude of variance than the 130‐member multimodel ensemble (Figure 1d), which further suggests the ensemble size dependency of the variance amplitude. If we simply select the 10 members from each individual model (or randomly out of 130 members), all seven models exhibit similar spatial patterns and strengths of the SIC‐driven variability (Figure S1).

To quantify the relationship between ensemble size and the estimate of Arctic SIC‐driven variance, we calculate the SIC‐driven variance for the Arctic‐averaged (65–90°N) SLP as a function of ensemble size (Figure 3a). For each ensemble size, we randomly select 10,000 samples (or the maximum possible samples if smaller than 10,000) out of 130 members without replacement. For example, when the ensemble size is set to 30, we randomly select 30 members (out of 130 members) 10,000 times to obtain 10,000 SIC‐driven variances. The mean value of these 10,000 SIC‐driven variances is shown in Figure 3 as solid line with 95% confidence level shaded. The SIC‐driven variance decreases exponentially toward ~0.18 hPa^2^ as the ensemble size increases toward 130 members. The exponential decay in SIC‐driven variance is also shown in Arctic‐averaged surface air temperature (SAT) SIC‐driven variance (Figure 3b), whose changes due to Arctic sea ice variability mostly represent the near‐surface thermodynamical response that can influence Arctic and midlatitude climate (Deser et al., 2015, 2016). The SAT variance exponentially decreases to ~0.35 K^2^ with 130 members used. We also perform the same analysis considering the ratios to total variability for each variance component (Figure S2) and overall similar ensemble size dependency is found for SIC‐driven components. The analysis for the ratios shows ~1.5% and ~21% of total variance can be explained by the SIC‐driven components for SLP and SAT, respectively using 130 members (Figures S2c and S2g). The results also suggest that using insufficient ensemble members always leads to an overestimation of the Arctic SIC‐driven variability.

To investigate how many ensemble members are needed to reach potential convergence for the SLP SIC‐driven component, we use a (rather strict) criterion that the absolute value of the change when one more member considered is smaller than 10^−2^ (%), that is,|*a*_*n + 1*_ *− a*_*n*_|< *O*(10^−2^), where the threshold is chosen to be of the order of the overall residual ratios described in section 2.2, and *a*_*n*_ represents variance ratio estimated with *n* ensemble numbers after a polynomial fit with the form *a/n* ^*b*^ *+ c*. The resultant parameter *b* is about 1.0005, indicating that the SIC‐driven component is roughly proportional to 1/*n* as expected. Based on this fitted curve, potential convergence is reached for an ensemble size of 134 (104–148, 95th percentiles), suggesting that at least 100 ensemble members are required to separate the SIC‐driven SLP variability from the other components, in particular the internal atmospheric noise, in AGCM simulations during the 1979–2014 period. The same analysis on SAT shows that the SIC‐driven SAT variability appears to attain convergence with 90 (41–131, 95th percentiles) members, which is smaller than the estimation for SLP.

We also quantify the relationship between ensemble size and the SIC‐driven variance in the selected midlatitude domains in Europe, Eurasia, and North America (Figures 3c and 3d) to assess the possible remote influence of Arctic SIC variability. Based on 130 members, the estimates for SIC‐driven SLP variances are ~0.14 hPa^2^ (~1.4% to total variance) in Europe, ~0.08 hPa^2^ (~1.7%) in Eurasia, and ~0.02 hPa^2^ (~1.9%) in North America (Figure 3c), all of which are smaller than the Arctic counterpart (~0.18 hPa^2^, Figure 3a). Smaller SIC‐driven variances are also found for SAT in midlatitude (Figure 3d), compared to the Arctic one (Figure 3b). The results suggest that SIC‐driven variability remotely in midlatitude are weaker for both dynamical and thermodynamical variables than those in high latitudes. We also investigate the ensemble sizes required to reach potential convergence for SIC‐driven components in midlatitude. For all three regions selected, very similar thresholds (130±6) are found for SLP, but the thresholds for SAT increase by about 40 members compared to that of the Arctic SAT. In summary, more than 100 members are needed for dynamical variables and more than 80 for thermodynamical ones are needed to robustly estimate the SIC‐driven variances in midlatitude.

The variability considered so far includes the contributions of long‐term trends as well as the interannual variability. To quantify the contributions of trends, we repeat the variance decomposition after removing a quadratic trend (Q‐trend hereafter) by least square fit at each grid point. We remove Q‐trend because the Arctic sea ice loss in autumn and winter in the past decades show accelerating rate, which is apparently not linear but closer to a quadratic structure (Dirkson et al., 2017). Top row panels of Figure 4 present the variances with the Q‐trends for Arctic SIC (i.e., the boundary condition used in ALL), and SIC‐driven variability of SLP, SAT, and total precipitation (i.e., large‐scale precipitation plus convective precipitation). The contribution of the trend to the SIC‐driven variance is quantified using the difference ratios (bottom row panels) defined as the differences between the variances with Q‐trend and without Q‐trend divided by the total variances. The trend contribution to SIC‐driven SLP variability is overall small (Figure 4d). In contrast, trend contributions to SAT and total precipitation are much larger, accounting for 10–20% near the Labrador Sea, ~15% in the Greenland Sea and Sea of Okhotsk, and ~40% over the Barents‐Kara Seas (Figures 4f and 4h), where a large trend contribution to SIC forcing is also found (Figure 4b). Thus, a portion of SIC‐driven SAT and total precipitation variability can be interpreted as a direct response to local SIC trend. The results indicate that the trend plays a greater role in the local variance of the thermodynamical (e.g., SAT) and hydrological (e.g., total precipitation) variables than for the dynamical ones (e.g., SLP).

## 4 Summary and Discussions

This study uses coordinated large ensemble AGCM experiments to examine the impacts of the observed Arctic sea ice variability on the atmospheric circulation at interannual and longer timescales in boreal winter for the 1979–2014 period. The variance decomposition using ALL and SIC_clim_ ensembles separates the Arctic SIC‐driven variability from the internal atmospheric noise and the variability forced by external forcings such as GHG, global SST and Southern Hemisphere sea ice. The robustness for the estimation of SIC‐driven variability dependent on the ensemble size is examined using all 130 members from the seven AGCMs. The results show that ~1.5% of the total Arctic‐averaged SLP variance and ~21% for Arctic‐averaged SAT are accounted for by the Arctic SIC‐driven variance. The results further suggest that for dynamical and thermodynamical variables more than 100 and 40 members, respectively, are needed to separate SIC‐driven variability from other components, in particular the atmospheric internal noise, and using insufficient ensemble members always leads to overestimations of both dynamical and thermodynamical variables within and outside the Arctic Circle. The long‐term trend contribution has little influence on the SIC‐driven SLP variability, but it explains up to 40% near the Arctic sea ice margins for thermodynamical and hydrological variables.

Screen et al. (2014) suggested that ~50 members are needed to separate the Arctic sea ice‐forced atmospheric response from internal atmospheric noise for SLP, and ~26 for SAT. The larger ensemble sizes suggested by our results are possibly due to different experimental design and sea ice forcing. For example, the spatial distribution of our time‐varying Arctic SIC forcing includes long‐term trend and variability in multiple timescales, while Screen et al. (2014) only considered a step change in SIC forcing that mimics the effect of long‐term trend, which corresponds to a larger forcing than that in our study. Also, their study used one AGCM instead of seven. CMIP6 Polar Amplification Model Intercomparison Project suggested at least 100 members for coordinated experiments (Smith et al., 2019). Our finding (more than 100 members for SLP) is consistent with Polar Amplification Model Intercomparison Project's suggested ensemble size and offers further insight on the need of larger ensemble size to robustly estimate the atmospheric response to Arctic sea ice change.

Our analysis indicates that the sea ice variation only explains a small percentage of interannual variability in the AGCMs, in particular for dynamical variables, such as SLP (Figures 3c and S2c). However, this primarily reflects the dominance of internal atmospheric variability, as the SIC‐driven SLP variance explains more than or is comparable to the variance driven by SST, GHG, and other forcings in the regions where sea ice variation is large, and ~75% over parts of northern Eurasia. Over the North Atlantic, Europe, and East Asia, the SIC‐driven variability is about 25–50% of that of the SST/GHG‐driven variability, indicating that the SIC variability exerts stronger impact locally in high‐latitude than remotely in midlatitude.

As recent studies reported controversial results for Arctic sea ice impacts on midlatitude variability (e.g., Overland et al., 2016; Blackport et al., 2019; Cohen et al., 2019; Peings, 2019; Screen & Blackport, 2019; Tyrlis et al., 2019), our findings reveal that such linkage could be overwhelmed by internal variability and other forcings due to the smallness of Arctic SIC‐driven variability, complicating our attempt to unravel the causality. However, the SIC‐driven variability is expected to increase in the future as the Arctic sea ice is projected to continue to retreat in the next 50 years in the CMIP5 simulations (Stroeve et al., 2012; Wang & Overland, 2012). A recent study showed that the atmospheric responses to Arctic sea ice changes only become stronger after middle of 21st century, while in late 20th century those are very weak (Sun et al., 2018). Therefore, future Arctic SIC‐driven variability may strongly affect midlatitude climate. In addition, the ocean dynamics could amplify the Arctic‐midlatitude linkage (e.g., Deser et al., 2015, 2016). An interactive ocean could enhance the remote impacts of Arctic SIC‐driven variability on midlatitudes. Coordinated future Arctic sea ice forcing and atmosphere‐ocean‐sea ice coupled experiments are needed to investigate these potentials. Also, the relative importance of the SIC‐driven variability could be dependent on the timescale, while this study only focuses on interannual and longer timescales. For example, the SIC‐driven variability at interannual or decadal timescales may be larger than those in subseasonal timescale. Finally, we cannot rule out the possibilities that the current generation AGCMs are not sensitive enough to boundary SIC forcings and thus largely affected by the internal noise (Screen et al., 2018), or the interdependency issue of model selection (Boé, 2018; Knutti et al., 2013). Therefore, a constraining approach based on the observational and reanalysis data sets is also desirable.

## Supporting information



Supporting Information S1

Figure S1

Figure S2
