# Supplementary material for: Quantification of the Arctic Sea Ice‐Driven Atmospheric Circulation Variability in Coordinated Large Ensemble Simulations
Source: Geophys Res Lett. 2020 Jan 17;47(1):e2019GL085397. doi: 10.1029/2019GL085397 (PMC7375043; doi:10.1029/2019GL085397)
Supplement: Supplementary file 1 — Supporting Information S1 [file GRL-47-e2019GL085397-s001.pdf]

Supporting Information for

Quantification of the Arctic Sea Ice-Driven Atmospheric Circulation Variability in Coordinated Large Ensemble Simulations

Yu-Chiao Liang[1*], Young-Oh Kwon[1], Claude Frankignoul[1,2], Gokhan Danabasoglu[3], Stephen Yeager[3], Annalisa Cherchi[4,5], Yongqi Gao[6,7], Guillaume Gastineau[2], Rohit Ghosh[8], Daniela Matei[8], Jennifer V. Mecking[9,10], Daniele Peano[4], Lingling Suo[6], and Tian Tian[11]

[1]Woods Hole Oceanographic Institution, Woods Hole, Massachusetts, U.S.A.

[2]Sorbonne Université, CNRS/IRD/MNHN, UMR LOCEAN, Paris, France.

[3]National Center for Atmospheric Research, Boulder, Colorado, U.S.A.

[4]Fondazione Centro Euro-Mediterraneo sui Cambiamenti Climatici, Bologna, Italy.

[5]Istituto Nazionale di Geofisica e Vulcanologia, Bologna, Italy.

[6]Nansen Environmental and Remote Sensing Center and Bjerknes Center for Climate Research, Bergen 5006, Norway.

[7]Nansen-Zhu International Research Center, Institute of Atmospheric Physics, Chinese Academy of Sciences, 100029 Beijing, People's Republic of China.

[8]Max Planck Institute for Meteorology, Hamburg, Germany.

[9]Ocean and Earth Science, National Oceanography Centre Southampton, University of Southampton, Southampton, United Kingdom.

[10]National Oceanography Centre Southampton, Southampton, United Kingdom.

[11]Danish Meteorological Institute, Copenhagen, Denmark.

*Correspondence to Yu-Chiao Liang (yliang@whoi.edu)

Contents of this file:

- Section S1.
- Table S1.
- Figures S1 and S2.

Introduction:

The text, table and figures in this supporting information complement the results shown in the main text.

- Section S1. Brief descriptions of Figures S1 and S2.
- Table S1. Summary of the AGCMs used in this study.
- Figure S1. The SIC-driven variability using 10 members in each model.
- Figure S2. The relationships between ensemble size and ratios of each variance component to total variance.

Section S1.

Fig. S1 shows the spatial maps of SLP SIC-driven variability of individual AGCM using only ten members in order to compare with results shown in Fig. 2. We also randomly chose 10 members out of 130 members across seven AGCMs (Fig. S2n) to show the strength of SIC-driven is largely model-independent.

Fig. S2 shows the quantification of the relationship between ensemble size and the portion of total variance explained by the Arctic-averaged (65°N-90°N) SIC-driven variability and the other components. For SLP, the contribution of internal atmospheric noise increases to more than 90% as the ensemble size becomes 15 (Fig. S2a). Correspondingly, the SST/GHG-driven variability exponentially decreases to ~10% (Fig. S2b) with 15 members, while the SIC-driven variability decreases to ~15% (Fig. S2c). At the same time, the covariance (multiplied by -1 for comparison to others) decreases to ~15%, while the residual variances remain very small (dashed lines near zero in Fig. S2d). Using 130 member, the SIC-driven variability is only ~1.5%. The total variance estimate remains nearly constant and has very little dependence on the ensemble size (not shown). Therefore, the variance is misleadingly attributed to the SST/GHG or SIC forcing instead of the internal variability when using small ensemble size. The same analysis for SAT is shown in Figs. S2e-h.

References:

Bentsen, M., Bethke, I., Debernard, J. B., Iversen, T., Kirkevåg, A., Seland, Ø., ... & Kristjánsson, J. E. (2013). The Norwegian earth system model, NorESM1-M—Part 1: Description and basic evaluation of the physical climate. *Geoscientific Model Dev*elopment, *6*(3), 687-720.

Cherchi, A., Fogli, P. G., Lovato, T., Peano, D., Iovino, D., Gualdi, S., ... & Navarra, A. (2019). Global Mean Climate and Main Patterns of Variability in the CMCC-CM2 Coupled Model. *Journal of Advances in Modeling Earth Systems*, *11*(1), 185-209.

EC-Earth (2019). The EC-Earth3 Earth System Model for the Climate Model Intercomparison Project 6. *Geoscientific Model Development Discussion*, in preparation. http://www.ec-earth.org/cmip6/ec-earth-in-cmip6/.

Gettelman, A., Mills, M. J., Kinnison, D. E., Garcia, R. R., Smith, A. K., Marsh, D. R., Tilmes, S., Vitt, F., Bardeen, C. G., McInerny, J., Liu, H.-L., Solomon, S. C., Polvani, L. M., Emmons, L. K., Lamarrque, J.-F., Richter, J. H., Glanville, A. S., Bacmeister, J. T., Philips, A. S., Neale, R. B., Simpson, I. R., DuVivier, A. K., Hodzic, A., & Randel, W. J. (2019). The Whole Atmosphere Community Climate Model version 6 (WACCM6). *Journal of Geophysical Research*.

Hourdin, F., Rio, C., Grandpeix, J.-Y., Madeleine, J.-B., Cheruy, F., Rochetin, N., Musat, I., Idelkadi, A., Fairhead, L., Foujols, M.-A., Mellul, L., Traore, A.-K., Dufresne, J.-L., Boucher, O., Lefebvre, M.-P., Millour, E., Vignon, E., Jouhaud, J., Diallo, F. B., Lott, F., Caubel, A., Meurdesoif, Y., & Ghattas, J. (2019), LMDZ-6A : the improved atmospheric component of the IPSL coupled model. *Journal of Advances in Modeling Earth Systems* (submitted, doi: http://www.lmd.jussieu.fr/~hourdin/TMP/ARTICLES_LMDZ_ENCOURS/LM

DZ6A/LMDZ6A_v11.pdf).

Müller, W. A., Jungclaus, J. H., Mauritsen, T., Baehr, J., Bittner, M., Budich, R., ... & Ilyina, T. (2018). A Higher‐resolution Version of the Max Planck Institute Earth System Model (MPI‐ESM1. 2‐HR). *Journal of Advances in Modeling Earth Systems*, *10*(7), 1383-1413.

Stevens, B., Giorgetta, M., Esch, M., Mauritsen, T., Crueger, T., Rast, S., ... & Brokopf, R. (2013). Atmospheric component of the MPI‐M earth system model: ECHAM6. *Journal of Advances in Modeling Earth Systems*, *5*(2), 146-172.

Walters, D. N., Baran, A., Boutle, I., Brooks, M. E., Furtado, K., Hill, P., ... & Sanchez, C. (2017). The Met Office Unified Model Global Atmosphere 7.0/7.1 and JULES Global Land 7.0 configurations, *Geoscientific Model Development*.

Table S1. Summary of the AGCMs used in this study

Model Name	CESM2-WACCM6	LMDZOR6	NorESM2-CAM6	EC-Earth3	CMCC-CM2-HR4	ECHAM6.3	HadGEM3	Multi-model ensemble
**Institution**	WHOI-NCAR	LOCEAN-IPSL	NERSC	DMI	CMCC	MPI-M	UoS	--
**Horizontal resolution (lat x lon)**	0.95° x 1.25° (~100 km)	1.26° x 2.5° (~150 km)	0.94° x 1.25° (~100 km)	T255 (~80 km)	0.9° x 1.25° (~100 km)	T127 (~100km)	0.83° x 0.55° (~60 km)	Interpolated to 0.95° x 1.25° (~100 km)
**# of vertical levels (top level)**	70 (0.001 hPa)	79 (0.01 hPa)	32 (3.4 hPa)	91 (0.01 hPa)	30 (2 hPa)	95 (0.01hPa)	85 (85 km)	--
**# of ensemble members**	30	30	20	20	10	10	10	130
**Adjustment of SST/SIC**	Yes	Yes	Yes	Yes	No	Yes	No	--
**CMIP6 External Forcing used**	CMIP6	HighResMIP	CMIP6	CMIP6	HighResMIP	CMIP6	HighResMIP	--
**Reference**	Gettelman et al. (2019)	Hourdin et al. (2019)	Bentsen et al. (2013)	EC-Earth (2019) Thomas et al. (2019)	Cherchi et al. (2018)	Stevens et.al.(2013) Mueller et. al. (2018)	Walters et al. (2017)	--

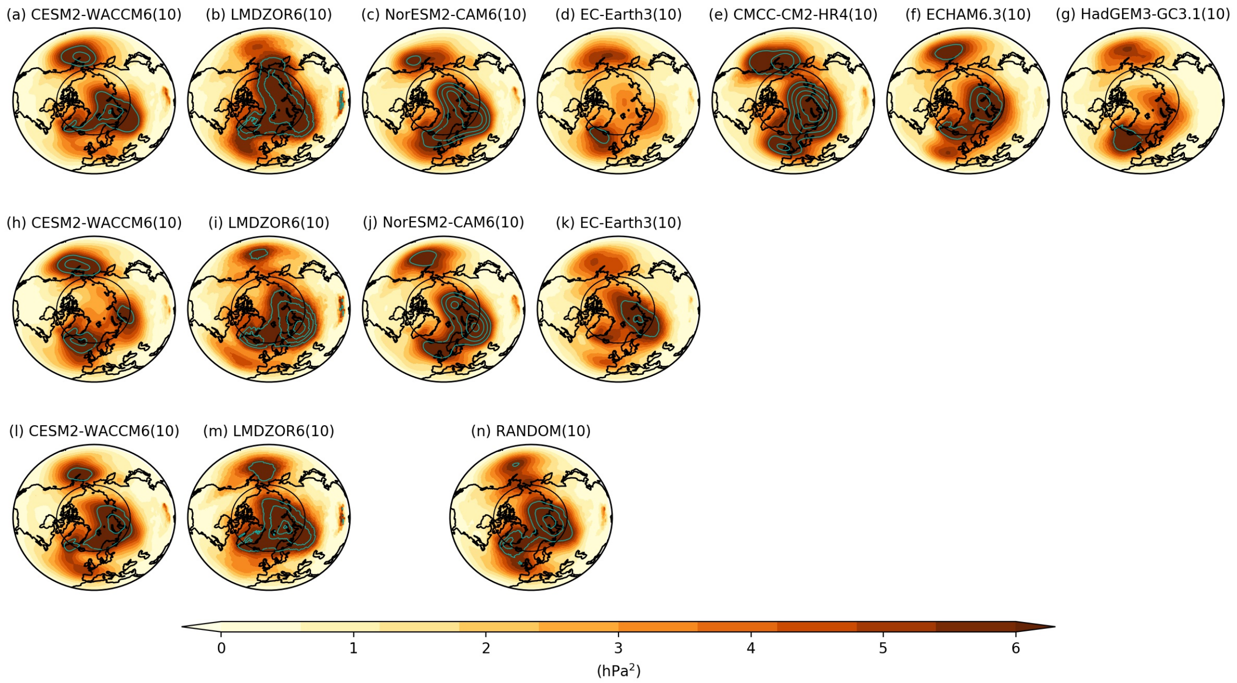

Figure S1. Arctic SIC-driven variance of DJF SLP using first ten members for (a) CESM2-WACCM6, (b) LMDZOR6, (c) NorESM2-CAM6, (d) EC-Earth3, (e) CMCC-CM2-HR4, (f) ECHAM6.3, and (g) HadGEM3-GC3.1. (h)-(k) The same as (a)-(d) but using second ten members. (l)-(m) The same as (a)-(b) but using third ten members. (n) The same as others but using 10 members randomly selected out of 130 members across seven AGCMs. The number in the parenthesis denotes ensemble size used. The cyan contour lines denote values larger than 6 hPa^2 with interval 2 hPa^2. The black circle corresponds to 65°N to denote the Arctic Circle.

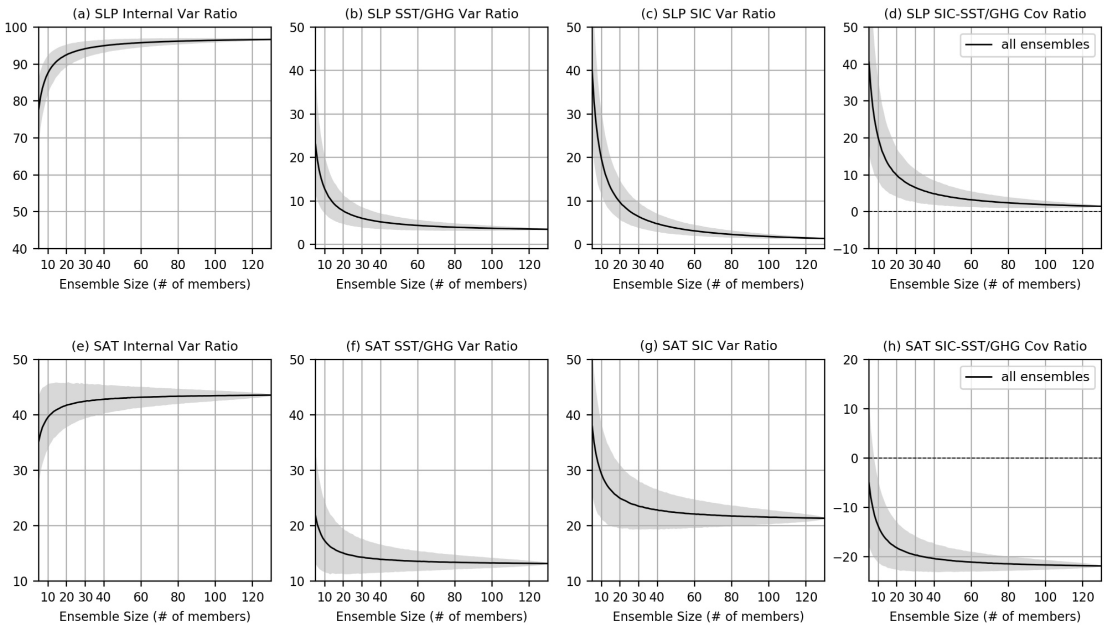

Figure S2. Ensemble size dependency for the ratio of estimated variances for each component to the total variance in percentage. The top row is for the Arctic Circle-averaged (65°N-90°N) DJF SLP decomposed into (a) internal atmospheric variability, (b) SST/GHG-driven variability, (c) SIC-driven variability, and (d) covariability between the SIC-driven and SST/GHG-driven components. For each given ensemble size, the ensemble members are randomly sampled without replacement 10,000 times. The color shadings indicate the 95-percentile range from 10,000 random selection and the average is plotted with the solid curves. (e)-(h) The same as (a)-(d) but for the near-surface air temperature in the same domain. The dashed lines in (d) and (h) are ratios for the residual components, which the 95-percentile ranges are also labeled but too small to be shown with the scale used here.
