# Supplementary figures and images for: Quantification of the Arctic Sea Ice‐Driven Atmospheric Circulation Variability in Coordinated Large Ensemble Simulations
Source: Geophys Res Lett. 2020 Jan 17;47(1):e2019GL085397. doi: 10.1029/2019GL085397 (PMC7375043; doi:10.1029/2019GL085397)

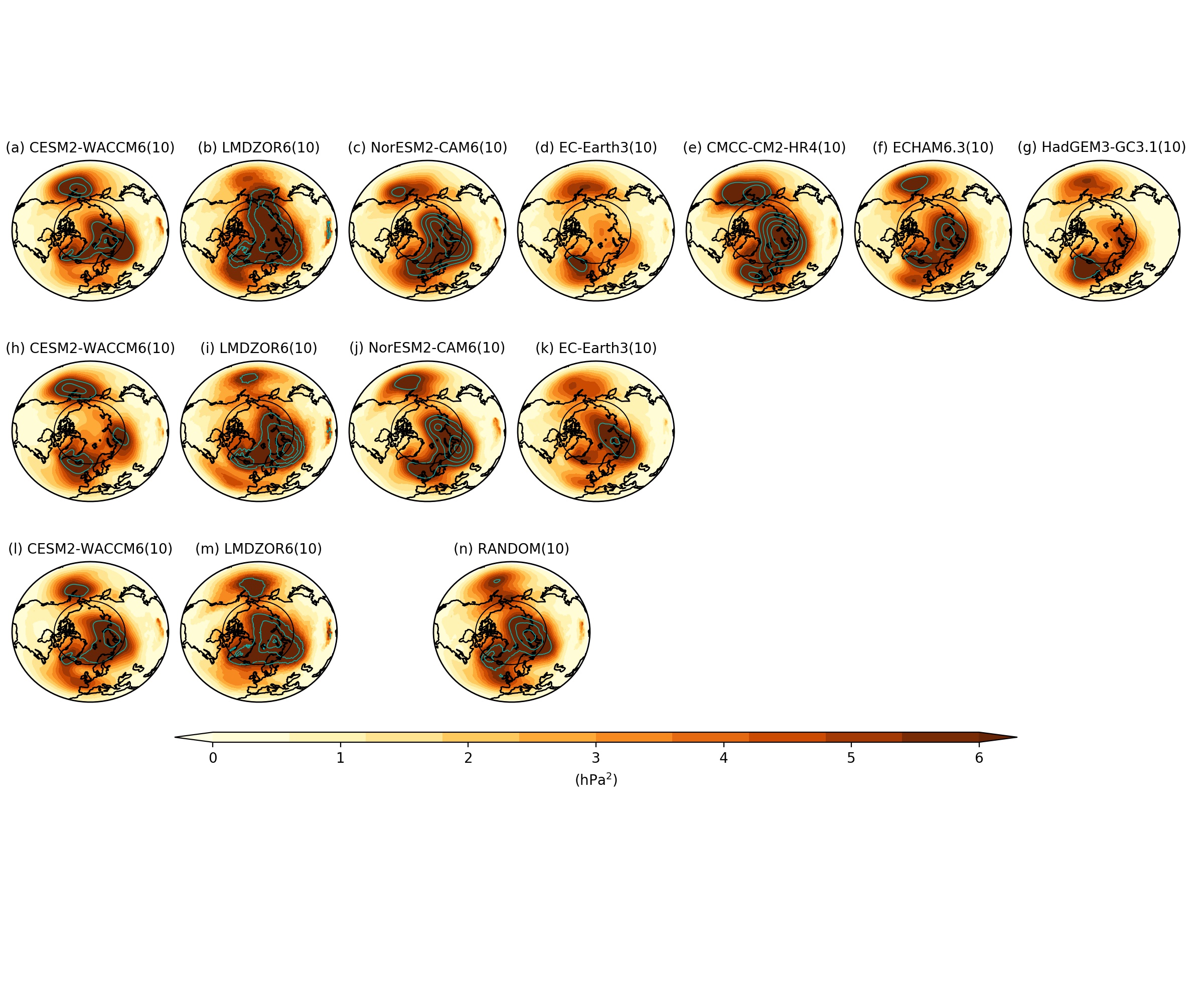

Supplement: Supplementary file 2 — Figure S1 [file GRL-47-e2019GL085397-s002.jpg]

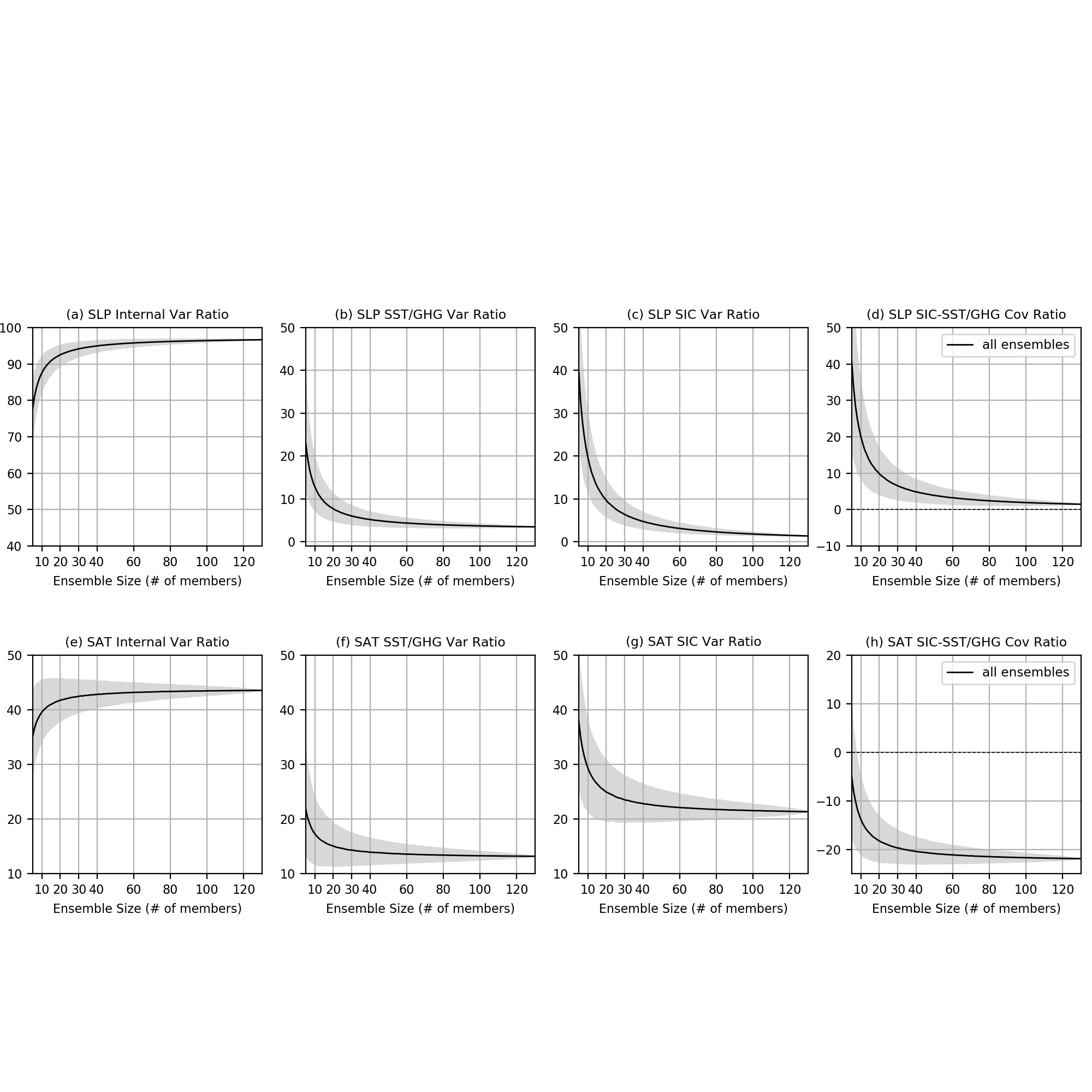

Supplement: Supplementary file 3 — Figure S2 [file GRL-47-e2019GL085397-s003.jpg]
